# A Systematic Review and Comprehensive Analysis of *mcr* Gene Prevalence in Bacterial Isolates in Arab Countries

**DOI:** 10.3390/antibiotics13100958

**Published:** 2024-10-11

**Authors:** Mouayad Zuheir Bakleh, Muhammad Kohailan, Muhammad Marwan, Abdallah Alhaj Sulaiman

**Affiliations:** 1 Division of Genomics and Precision Medicine, College of Health and Life Sciences, Hamad Bin Khalifa University, Education City, Qatar Foundation, Doha P.O. Box 34110, Qatar; 2 Qatar Precision Health Institute, Qatar Foundation, Doha P. O. Box 5825, Qatar; 3 Division of Biopsychology and Neuroscience, College of Health and Life Sciences, Hamad Bin Khalifa University, Education City, Qatar Foundation, Doha P.O. Box 34110, Qatar; 4 Division of Biological and Biomedical Sciences, College of Health and Life Sciences, Hamad Bin Khalifa University, Education City, Qatar Foundation, Doha P.O. Box 34110, Qatar

**Keywords:** *mcr* genes, colistin, antimicrobial resistance, Arab countries

## Abstract

Background: The resurgence of colistin has become critical in combating multidrug-resistant Gram-negative bacteria. However, the emergence of mobilized colistin resistance (*mcr*) genes presents a crucial global challenge, particularly in the Arab world, which includes regions with unique conditions and ongoing conflicts in some parts. Methods: To address this issue, a systematic review was conducted using multiple databases, including Cochrane, PubMed, Scopus, Web of Science, and Arab World Research Source. Results: A total of 153 studies were included, revealing substantial heterogeneity in the prevalence of *mcr* genes across 15 Arab countries, with notable findings indicating that Egypt and Lebanon reported the highest number of cases. The analysis indicated that the most prevalent sequence types were ST10, ST101, and ST1011, all of which are *Escherichia coli* strains linked to significant levels of colistin resistance and multiple antimicrobial resistance profiles. Conclusions: By analyzing the diverse findings from different Arab countries, this review lays a critical foundation for future research and highlights the necessity for enhanced surveillance and targeted interventions to address the looming threat of colistin resistance in the region. Systematic review registration: PROSPERO CRD42024584379.

## 1. Introduction

Colistin, or polymyxin E, is a decades-old antibiotic that was sidelined due to its nephrotoxicity. Nevertheless, it has been reintroduced due to the emergence of multidrug-resistant (MDR), including carbapenem-resistant, Gram-negative bacteria such as *E. coli*, *Klebsiella pneumoniae*, *Salmonella* spp., *Acinetobacter baumannii*, *Pseudomonas aeruginosa*, and others [1,2]. Colistin disrupts the bacterial outer membrane by binding to the lipid A component of lipopolysaccharides in the Gram-negative strains, leading to cell lysis [3]. However, resistance mechanisms have evolved, primarily through modifications of lipid A that reduce colistin binding affinity. These modifications are frequently mediated by the mobilized colistin resistance (*mcr*) genes, which encode a phosphoethanolamine transferase enzyme that adds a phosphoethanolamine group to the lipid A, leading to colistin resistance [4]. The first *mcr* gene, *mcr-1*, was identified in 2015 in an *E. coli* isolate from a pig in China, marking a significant breakthrough in understanding colistin resistance [5]. Also, in 2016, the *mcr-1* gene was first identified in a human isolate in the United States, from a patient with no history of international travel for at least 1 year and no livestock exposure [6]. Since then, multiple variants of *mcr* genes, including *mcr-2* to *mcr-10*, have been identified across diverse geographical regions and bacterial species [7,8]. These genes are often located on plasmids, facilitating their vertical or horizontal transfer between the same or different bacterial species and contributing to their rapid global dissemination [8]. These variants differ in their genetic sequences but share a common function: the modification of the bacterial lipid A, which reduces colistin’s ability to bind and eliminate the bacteria. This variation allows for the rapid global spread of resistance in clinical and agricultural settings [9].

Globally, antimicrobial resistance (AMR) genes, including *mcr* genes, are reported in various clinical and environmental sectors, contributing to 4.95 million deaths in 2019, with projections of 10 million annual deaths by 2050 [10,11]. Infections caused by colistin-resistant pathogens result in prolonged illness and recovery times, leading to increased suffering and higher medical costs [12]. Healthcare-associated infections caused by *mcr*-carrying isolates are associated with higher morbidity and mortality rates. The presence of *mcr* genes often indicates the presence of other resistant genes, leading to resistance to multiple antibiotics, including carbapenems, leaving clinicians with fewer therapeutic options and making infections difficult to treat effectively [10,11].

The use of colistin in veterinary medicine, particularly in food-animal production, contributes to the spread of resistance from animals to humans, complicating efforts to prevent and control infections in both sectors [10]. The use of colistin as a growth promoter in animals to meet protein demands at affordable prices has accelerated resistance. Additionally, the lack of regulations and the bypassing of specific instructions for colistin use in several countries have exacerbated the issue of colistin resistance [11].

Despite these alarming trends of increased prevalence of *mcr* genes and high mortality rates due to AMR, a major gap in the current research is the lack of detailed data on the prevalence and distribution of *mcr* genes in specific regions [13], particularly in the Arab world, where healthcare systems, agricultural practices, and regulatory frameworks differ significantly from other parts of the globe. There is a limited understanding of how these factors contribute to the spread of *mcr*-mediated resistance in this region. The Arab world, with its unique healthcare infrastructure, agricultural practices, regulatory frameworks, and ongoing conflicts in some of its parts, presents distinct pathways that facilitate the dissemination of *mcr* genes, leading to colistin resistance.

This systematic review addresses several knowledge gaps by focusing on the prevalence of *mcr* genes in animal, clinical, and environmental isolates within Arab countries. It examines the distribution, sources, and implications of *mcr*-carrying bacteria in this region, identifying gaps in existing data for each country. The primary objective of this review is to emphasize findings from current studies and assess the relative distribution of *mcr* genes across Arab countries. The analysis provided in this review can be used as baseline data to understand the current situation in each Arab country. Understanding the regional dynamics of *mcr* gene prevalence contributes to the global effort to combat antibiotic resistance and protect public health.

## 2. Materials and Methods

### 2.1. Literature Search

This systematic review follows the Preferred Reporting Items for Systematic Reviews and Meta-Analyses (PRISMA) guidelines. The protocol was registered with the International Prospective Register for Systematic Reviews (PROSPERO CRD42024584379), and the protocol has been described for this systematic review [14]. The article search was conducted on 23 June 2024, using the following databases, all accessed on the same date: Cochrane (https://www.cochrane.org), PubMed (https://pubmed.ncbi.nlm.nih.gov), Scopus (https://www.scopus.com), Web of Science (https://webofscience.com), and Arab World Research Source (https://www.ebsco.com). Medical Subject Headings (MeSH) were utilized to define synonyms for the *mcr* and Arab countries’ terminologies. The complete search strategy and terms used to collect the studies are detailed in Supplementary File S1, sheet 1.

### 2.2. Inclusion and Exclusion Criteria

For the inclusion criteria, (1) we selected studies involving bacterial isolates from any source in the Arab region. (2) These studies had to report the presence of *mcr* genes (*mcr-1*, *mcr-2*, etc.) detected through molecular methods (PCR, sequencing, etc.). (3) We included primary research studies such as cross-sectional studies, letters, cohort studies, case–control studies, and surveillance reports. (4) The geographic scope was limited to countries within the Arab region, including Algeria, Bahrain, Comoros, Djibouti, Egypt, Iraq, Jordan, Kuwait, Lebanon, Libya, Mauritania, Morocco, Oman, Palestine, Qatar, Saudi Arabia, Somalia, Sudan, Syria, Tunisia, United Arab Emirates, and Yemen. (5) Only studies published in English or Arabic, or those with available translations, were considered. (6) The publication date had to be after 2015, as *mcr* genes were only discovered following that year. For the exclusion criteria, (1) we excluded studies not conducted within the specified Arab countries or those focusing exclusively on non-Arab populations or geographical regions. (2) Studies that did not report on the presence of *mcr* genes or focused on other mechanisms of colistin resistance were also excluded. (3) We did not include reviews, editorials, commentaries, conference abstracts, and non-original research articles. (4) Additionally, studies lacking clear data on *mcr* gene prevalence, those with incomplete reporting, or those with insufficient detail on methodology or results were excluded. (5) Studies not published in English or Arabic without available translations, as well as duplicate publications or redundant data from the same study or dataset, were also excluded.

### 2.3. Study Selection and Data Retrieval

Articles retrieved from the search strategy were initially imported into EndNote 20.4 (Bld18004) to remove duplicates and then exported into an Excel spreadsheet containing key details such as year of publication, title, abstract, and other pertinent information for further evaluation (Supplementary File S1, sheet 2). The screening process was conducted by one author and independently reviewed by the same author. This process was carried out in two stages: first, the titles and abstracts were reviewed to exclude publications that did not meet the predefined criteria (Supplementary File S1, sheet 3); and second, the full texts of the selected articles were assessed to determine the final set of publications eligible for data extraction and analysis (Supplementary File S1, sheet 4).

Data extraction included information on country of origin, source, isolate type, number of isolates, gene modifications associated with colistin resistance, resistance profile, MIC values for colistin, sequencing details, accession numbers, ST types, AMR genes, detected plasmids, virulence genes, and year of publication. Additional details, such as phylogroup and FimH type, were also recorded. A comprehensive list of the extracted information is available in Supplementary File S1, sheet 5. The selected articles underwent a final review for inclusion and exclusion criteria by two independent authors to ensure consistency and accuracy in the selection process. The data were initially extracted manually by one author and subsequently reviewed independently by a different author. The retrieved data were analyzed according to the PICOS format as follows: Population included bacterial isolates collected from all sources within the Arab region. Intervention or exposure involved colistin exposure and the presence of *mcr* genes detected in these bacterial isolates. Comparison was made across different countries and/or over time. Outcomes considered included prevalence rates, distribution of bacterial species, co-occurrence with other AMR genes, and temporal trends in *mcr* gene prevalence. Study design comprised any primary research.

The data extraction process involved identifying and categorizing outcomes such as prevalence rates, distribution of bacterial species, co-occurrence with other AMR genes, and temporal trends in *mcr* gene prevalence. All relevant data compatible with these outcomes, regardless of measures, time points, or analyses, were sought from each study. If certain results were not directly reported, the data were cross-referenced with other sections of the study or Supplementary Materials. The final selection of results for each outcome was based on consistency and completeness across studies.

### 2.4. Data Analysis and Visualizations

Data on the distribution of *mcr* types among Arab countries were analyzed using programing language libraries that were selected due to their ability to analyze large datasets, including geopandas, pandas, NumPy, and Matplotlib. A shapefile of world administrative boundaries was loaded with geopandas, and the dataset was filtered to include only Arab countries based on their ISO_A3 codes. The distribution data were integrated with the geographic data, and country sizes were adjusted according to predefined scaling factors to enhance visualization. A heatmap was generated using Matplotlib to represent the distribution of *mcr* types among these countries.

The same packages were used to analyze and visualize source data categorization by country. The country names were standardized by stripping spaces. A dictionary was created to categorize sources into animal, clinical, and environmental groups. A function was applied to categorize each source, and uncategorized sources were redistributed manually.

A directed network graph was constructed using ST1011, ST101, and ST10 to visualize their distribution across various countries. The graph was generated using the NetworkX and Matplotlib packages. Nodes represented the STs and the countries, with edges indicating the number of occurrences, weighted by count, and labeled with percentages.

Pandas, Seaborn, and Matplotlib were used to visualize MIC values by ST type. The ‘MIC’ column was ensured to be numeric, and infinite values were replaced with NaN. The dataset was filtered to include only the top 10 most frequent ST types among the reported Arab countries. The highest MIC value for each of these ST types was calculated and visualized.

Similar to the method described by [15], we calculated the minimum spanning tree of a directed graph constructed from asymmetric distances between ST types. This analysis utilized pandas, NumPy, Matplotlib, and NetworkX to visualize plasmid distribution among selected ST types [15]. Plasmid data were standardized, filtered, and expanded to count occurrences. The top 10 frequent plasmids were identified, and a pivot table was created. Then, the minimum spanning figure was generated for visualization.

Antibiotics were categorized into families and mapped to their corresponding families. The data were processed to count occurrences by family and country and visualized using a heatmap with Matplotlib. The dataset was organized into a DataFrame, normalized based on profile numbers from all the studied countries, and subjected to logarithmic transformation to handle large differences. A radar chart was generated to compare antibiotic families across grouped countries. Statistical analyses, including *t*-tests and ANOVA, were conducted to evaluate differences and obtain *p*-values.

The analysis of virulence gene data was performed using pandas, NumPy, and Matplotlib packages. The data were preprocessed by removing rows with missing or non-informative entries. A dictionary was created to count the occurrences of each virulence gene by country. Genes occurring in at least two countries were retained for analysis. The processed data were visualized using a heatmap generated with Matplotlib.

A Sankey diagram was created using the Plotly package to visualize the flow of sequence types across Arab countries. The diagram represents connections between countries and STs, with percentages indicating the frequency of occurrences.

### 2.5. Bioinformatics Analysis

For each study, the publicly available whole-genome sequencing files of the *E. coli* isolates were downloaded from NCBI using the corresponding accession numbers. The *E. coli* genome, K-12 MG1655 (GenBank accession: CP014225.1), was used as a reference for the genome sequence alignments. First, individual sample alignments were performed using the Burrows–Wheeler Alignment tool (BWA, version 0.7.18, [16]). Then, bcftools (version 1.20, [17]) was used for variant calling and consensus sequence generation. After merging all resulted consensus sequences, multiple-sample alignment was performed using MAFFT (version 7.526, [18]), and the phylogenetic tree was generated using iqtree2 (version 2.3.5, [19]). Finally, the generated tree was visualized in R using ggtree library (version 3.10.1, [20]).

### 2.6. Assessment of Bias Risk

To assess and visualize the risk of bias across multiple studies, we utilized pandas and plotly.express packages. The dataset containing the risk of bias assessments was loaded from (Supplementary File S1, sheet 6). We cleaned the data by stripping any leading or trailing whitespaces and converting entries to lowercase. Risk ratings were then mapped to numerical values: ‘low’ to 1, ‘moderate’ to 2, and ‘high’ to 3. The cleaned data were transformed into a long format suitable for visualization. An interactive heatmap was created using plotly.express, with a custom color scale to represent different risk levels. The resulting figure was saved as an HTML file for easy sharing and interactive exploration (Supplementary File S2). The risk of bias analysis was based on five possible domains: D1: Selection Bias: Methods of isolate selection; random or convenience sampling. D2: Performance Bias: Handling and testing of isolates; blinding of laboratory personnel. D3: Detection Bias: Standardization and validation of *mcr* gene detection methods. D4: Attrition Bias: Completeness of outcome data; reporting of missing data. D5: Reporting Bias: Reporting of all predefined outcomes; selective outcome reporting. The inputs under each domain led to the generation of graphical representations of “Low risk of bias”, “Moderate concerns”, or “High risk of bias” [21].

## 3. Results

### 3.1. Study Selection

**Figure 1:**
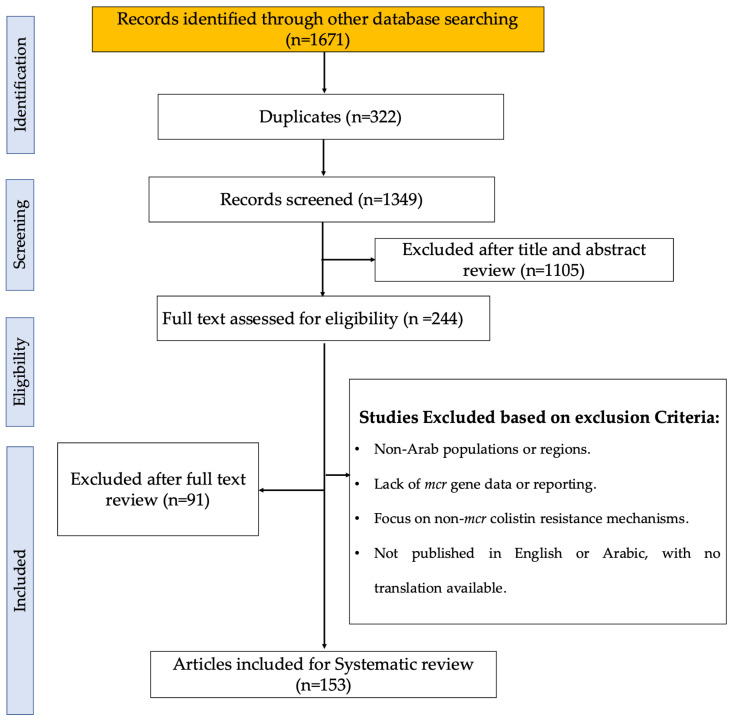
PRISMA flow diagram of the study selection process, illustrating the identification, screening, eligibility, and inclusion stages. The diagram details the number of records retrieved from databases, the number of duplicates removed, and the articles screened, excluded, and included in the final analysis [22].

A comprehensive database search identified 1671 records. After removing duplicates, 1349 articles remained and were subjected to initial screening. Out of these, 1105 records were excluded based on the title and abstract review. The remaining 244 records were then assessed for eligibility based on their full-text content. Post-assessment, 91 articles were excluded based on the exclusion criteria. Ultimately, 153 articles met all the inclusion criteria and were included in the systematic review (Figure 1), providing the dataset for further analysis. The progress of publications related to *mcr* genes in different Arab countries from 2016 to 2024 is shown in Supplementary Figure S1A–O. The data reflect variability in the volume of research over time across these countries. Egypt exhibits the highest number of studies, particularly peaking between 2019 and 2021, where several *mcr* genes were reported, including *mcr-1*, *mcr-2*, *mcr-3*, *mcr-4*, *mcr-7*, *mcr-9*, and *mcr-10*. Lebanon follows with a notable increase in 2020–2021, recording genes such as *mcr-1*, *mcr-8*, and *mcr-9*. Algeria shows an increase with publications between 2019 and 2022, with findings of *mcr-1*, *mcr-5.1*, *mcr-3*, and *mcr-8*. Other countries like Tunisia displayed moderate activity in publications with *mcr-1* and *mcr-2* identified in 2016–2023, while Saudi Arabia shows notable peaks in 2021 and 2022 with *mcr-1*, *mcr-4*, *mcr-8*, and *mcr-9* reported. The UAE has *mcr-1* findings in 2016, 2022, 2023, and 2024. Qatar displayed publications in 2018, 2020, and 2022, with the detection of *mcr-1* and *mcr-9*. Meanwhile, countries such as Bahrain, Oman, and Libya have reported very few studies, indicating significant data gaps, with *mcr-1* being the predominant gene across the years (Supplementary Figure S1A–O). No studies were found reporting the presence of *mcr* genes in Arab countries that are not reported in Table 1.

### 3.2. Geographic and Bacterial Distribution of *mcr* Genes Across Arab Countries

**Figure 2:**
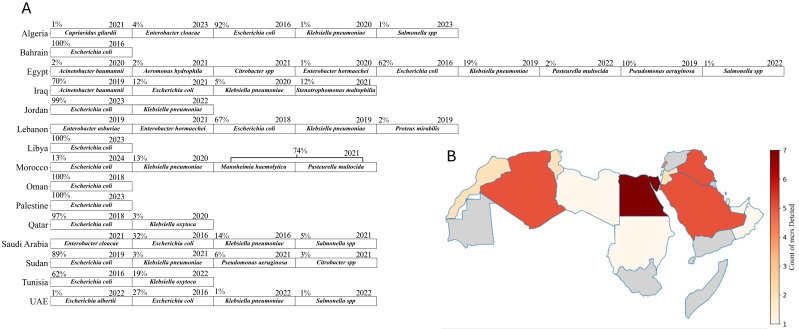
Distribution and diversity of *mcr*-positive bacterial species and *mcr* gene types across Arab countries. (**A**) The percentages indicate the proportion of each bacterial species among *mcr*-positive isolates within each country. When no percentage is shown, it means that the proportion was undetermined due to a lack of sufficient data. The year indicates the first recorded detection of the respective strain in each country. (**B**) A heatmap illustrating the count of different *mcr* gene types (e.g., *mcr-1*, *mcr-2*, *mcr-3*, etc.) reported in Arab countries. The intensity of the red color corresponds to the number of *mcr* types identified, with darker shades representing a higher diversity of *mcr* types. Countries shaded in gray indicate either no studies or studies that reported negative results for the *mcr* genes. Edited with BioRender.com.

The screening of studies from twenty-two Arab countries investigated the presence of *mcr* genes in different sources, including animal, environmental, and clinical. The countries included in this analysis are listed in the research strategy detailed in Supplementary File S1, sheet 1. Of these, fifteen countries reported the presence of *mcr* genes from various strains (Table 1 and Figure 2A). The data reveal significant variability in bacterial isolates and *mcr* gene prevalence across the Arab region. Egypt, which has the highest number of studies (53) to describe the presence of *mcr* genes, displays a diverse range of bacterial strains (nine strains), including *E. coli*, *K. pneumoniae*, and *P. aeruginosa*, with a broad spectrum of *mcr* genes detected (Figure 2A,B). Lebanon follows with a high report of *E. coli* isolates and a notable detection of *mcr-1.26* from environmental and clinical samples [23,24], although it reports fewer variants of *mcr* genes compared to Egypt (Figure 2A,B). Algeria and Tunisia have fewer studies and isolate detection compared to Egypt. Algeria shows a range of *mcr* genes, including *mcr-5.1* in *C. gilardii* [25] surpassing Tunisia in *mcr* gene diversity but lagging behind Egypt. Saudi Arabia and Qatar have smaller datasets, with *mcr-1* genes predominantly found in *E. coli*. Also, the UAE reports a high prevalence of *mcr-1* across various bacterial species, including *S.* Minnesota, *similar to Saudi Arabia*. Oman and Palestine have minimal data, with only a single isolate reported in Oman from a clinical source [26]. Jordan and Iraq exhibit broader *mcr* gene diversity, with Jordan reporting *mcr-10* in *E. coli* and Iraq showing a wide range of *mcr* types across multiple bacteria. Overall, Egypt and Lebanon have the most extensive and varied datasets (Table 1, Figure 2A,B).

**Table 1:** Summary of studies reporting *mcr* genes in bacterial isolates in the Arab countries. This table details the number of studies per country, the types and counts of bacterial isolates examined, and the *mcr* genes detected in isolates detected in the reported Arab countries.

Country	No. of Studies	Isolate Type and Count	MCR Detected	Reference
Egypt	53	*E. coli (327)*	*mcr-(1*, *2*, *3*, *4*, *9)*	[1,27,28,29,30,31,32,33,34,35,36,37,38,39,40,41,42,43,44,45,46,47,48,49,50,51,52,53,54,55,56,57,58,59,60,61,62,63,64,65,66,67,68,69,70,71,72,73,74,75,76,77,78]
		*K. pneumoniae (103)*	*mcr-(1*, *2*, *3*, *10)*	
		*P. aeruginosa (54)*	*mcr-(1*, *2*, *3*, *7)*	
		*E. hormaechei (6)*	*mcr-9*	
		*A. baumannii (13)*	*mcr-1*	
		*A. hydrophila (10)*	*mcr-(1*, *2*, *3)*	
		*Citrobacter freundii (1)*	*mcr-1*	
		*Salmonella (3)*	*mcr-1*	
		*P.multocida (12)*	*mcr-1*	
Lebanon	27	*E. coli (348)*	*mcr-(1*, *1.26)*	[23,24,79,80,81,82,83,84,85,86,87,88,89,90,91,92,93,94,95,96,97,98,99,100,101,102,103]
		*Proteus mirabilis (9)*	*mcr-1*	
		*K. pneumoniae (1)*	*mcr-8.1*	
		*E. hormaechei (1)*	*mcr-9*	
		*E. coli K. pneumoniae*, *E. Asburiae (163)*	*mcr-1*	
Algeria	16	*E. coli (67)*	*mcr-(1*, *1.5*, *3)*	[25,104,105,106,107,108,109,110,111,112,113,114,115,116,117,118]
		*K. pneumoniae (1)*	*mcr-8*	
		*C. gilardii (1)*	*mcr-5.1*	
		*E. cloacae (3)*	*mcr-1*	
		*S.enterica (1)*	*mcr-1*	
Tunisia	14	*E. coli (216)*	*mcr-(1*, *2)*	[119,120,121,122,123,124,125,126,127,128,129,130,131,132]
		*K. oxytoca (1)*	*mcr-1*	
Saudi Arabia	11	*E. coli (18)*	*mcr-1*	[133,134,135,136,137,138,139,140,141,142,143]
		*K. pneumoniae (8)*	*mcr-(1*, *8*, *8.1)*	
		*S. enterica (3)*	*mcr-(1*, *9)*	
		*E. coli and K. pneumoniae E. cloacae (27)*	*mcr-(1*, *4)*	
Qatar	6	*E. coli (36)*	*mcr-1*	[144,145,146,147,148,149]
		*Klebsiella oxytoca (1)*	*mcr-9*	
UAE	8	*E. coli (69)*	*mcr-1*	[133,150,151,152,153,154,155,156]
		*S.* Minnesota *(3)*	*mcr-1*	
		*E. albertii (2)*	*mcr-1*	
		*K. pneumoniae (2)*	*mcr-1*	
		*E. coli*, *S.* Minnesota, *E. albertii*, *K. pneumoniae (177)*	*mcr-1*	
Oman	1	*E. coli (1)*	*mcr-1*	[26]
Jordan	5	*E. coli (201)*	*mcr-(1*, *10)*	[157,158,159,160,161]
		*K. pneumoniae (1)*	*mcr-1*	
Palestine	1	*E. coli (25)*	*mcr-1*	[162]
Iraq	6	*A. baumannii (143)*	*mcr-(1*, *2*, *3)*	[163,164,165,166,167,168]
		*K. pneumoniae (11)*	*mcr-(1*, *2*, *4)*	
		*E. coli (25)*	*mcr-(1*, *2*, *3*, *4*, *5)*	
		*S. maltophilia (24)*	*mcr-1*	
Sudan	2	*E. coli (32)*	*mcr-1*	[169,170]
		*Citrobacter species (1)*	*mcr-1*	
		*K. pneumoniae (1)*	*mcr-1*	
		*P. aeruginosa(2)*	*mcr-1*	
Libya	1	*E. coli (4)*	*mcr-1*	[171]
Morocoo	3	*E. coli (1)*	*mcr-1*	[172,173,174]
		*K. pneumoniae (1)*	*mcr-8*	
		*M. haemolytica & P. multocida (6)*	*mcr-1*	
Bahrain	1	*E. coli (2)*	*mcr-1*	[133]

### 3.3. Diverse Source Origins and Key Sequence Types of mcr Genes in Arab Countries

**Figure 3:**
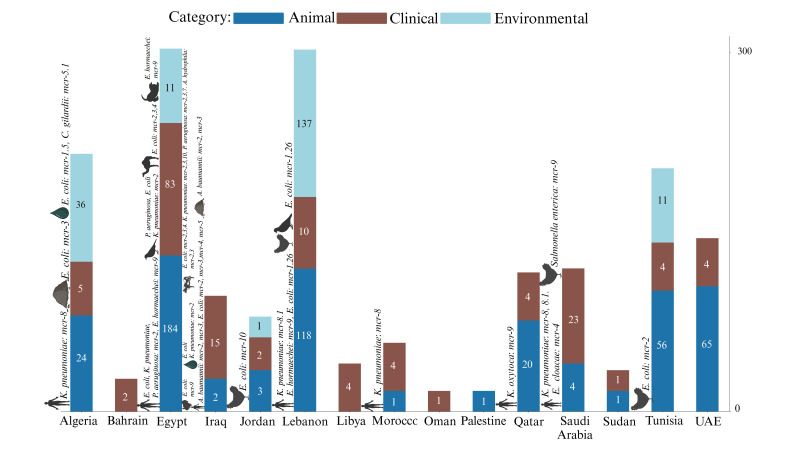
Source distribution and strain prevalence of *mcr*-harboring bacterial isolates. The figure reflects the Distribution of *mcr* gene-harboring bacterial isolates across different source categories (animal, clinical, environmental) in 15 Arab countries. The bars represent the number of samples from each source, with color coding indicating the category. Symbols above the bars represent the sources of *mcr* genes other than *mcr-1*, which is the most predominant and therefore not separately symbolized.

Egypt has a substantial focus on animal sources (184), which is significantly higher than Lebanon’s (118) animal sources, considering that Lebanon is mentioned in fewer studies. Lebanon, however, shows a larger environmental presence (137) compared to Egypt’s 11, indicating that Lebanon’s studies are more focused on environmental surveillance. Algeria has a balanced distribution across environmental (36) and animal (24) sources, but fewer in clinical (5) sources. However, there is a notable detection of *mcr-8* in clinical *K. pneumoniae* and *mcr-3* in *E. coli* isolated from soil samples, suggesting a more even spread of *mcr* genes across different sources. In contrast, Tunisia has a predominant animal source presence (56), similar to Egypt but with less variability of *mcr* genes with a notable report of *E. coli* (*mcr-2*) from chicken samples. Saudi Arabia emphasizes clinical sources (23) more than any other type of sources, highlighting significant clinical challenges with *mcr* genes such as clinical *K. pneumoniae* (*mcr-8*, *8.1*) and *E. cloacae* (*mcr-4*), as well as *S.* Minnesota with (*mcr-9*) isolated from chicken samples. Qatar shows a predominance of animal sources (20) and fewer clinical sources (4), reflecting a focus similar to Tunisia but with different *mcr* gene types like *K. oxytoca* (*mcr-9*) and an absence of environmental sources. The samples from Qatar vary across a range of sources, including clinical isolates, poultry farms, live bird markets, rectal swabs from humans (including children), pigeons, and retail chicken. The UAE also shows a significant animal source presence (65) but includes some clinical sources (4), with no *mcr* detection beside *mcr-1*. Jordan presents a more balanced distribution with animal (3), clinical (2), and environmental (1) sources, similar to Algeria but on a smaller scale, with *mcr* genes like *E. coli* (*mcr-10*) isolated from chicken samples. Iraq, with six studies, highlights significant clinical sources (15), indicating clinical challenges similar to Saudi Arabia, with *mcr* genes such as the ones harbored by *A. baumannii* (*mcr-2* and *mcr-3*) isolated from clinical and environmental samples. Sudan, with two studies, reports *E. coli* and other strains with (*mcr-1*) in both animal and clinical sources, showing a mix of reservoirs on a smaller scale. Countries with fewer studies, including Palestine (one study), Libya (one study), Morocco (three studies), and Bahrain (one study), show limited sources, suggesting lower prevalence or data gaps. Morocco reports *K. pneumoniae* (*mcr-8*) primarily in clinical sources, unlike the more varied distributions seen in other countries. The relatively high presence of sources in Egypt and Lebanon contrasts sharply with their near absence in many other countries, indicating potential gaps in reporting *mcr* genes in several sources elsewhere (Figure 3). This varied distribution of *mcr* gene types and source categories across countries emphasizes the need for tailored, region-specific surveillance and intervention strategies.

We further examined the distribution of specific sequence types within the region. From the data, we extracted around 150 sequence types representing various bacteria, primarily *E. coli*, across 12 Arab countries (Supplementary File S3). To focus on the most significant ones, we filtered the top 10 most frequently occurring sequence types and the top 10 most geographically distributed sequence types (Supplementary Figure S2). The intersection of these two groups revealed three prominent sequence types: ST101, ST1011, and ST10. The ST101 shows significant prevalence in Algeria, with a notable proportion also observed in United Arab Emirates, Egypt, and Lebanon. ST1011, on the other hand, is predominantly found in Lebanon and the UAE, with a smaller presence in Egypt, Qatar, and Tunisia. ST10 demonstrates the broadest distribution, being present across Algeria, Egypt, Lebanon, Oman, Qatar, Saudi Arabia, Tunisia, and the United Arab Emirates, with particularly high proportions in Lebanon and Algeria. This analysis complements the previous data, showing that while source types (clinical, environmental, animal) and specific *mcr* gene types vary across countries, the spread of certain sequence types like ST101, ST1011, and ST10 is extensive, requiring focused regional surveillance efforts (Supplementary Figure S3).

### 3.4. Minimum Inhibitory Concentration (MIC) Variability and Plasmid Associations of Key Sequence Types

**Figure 4:**
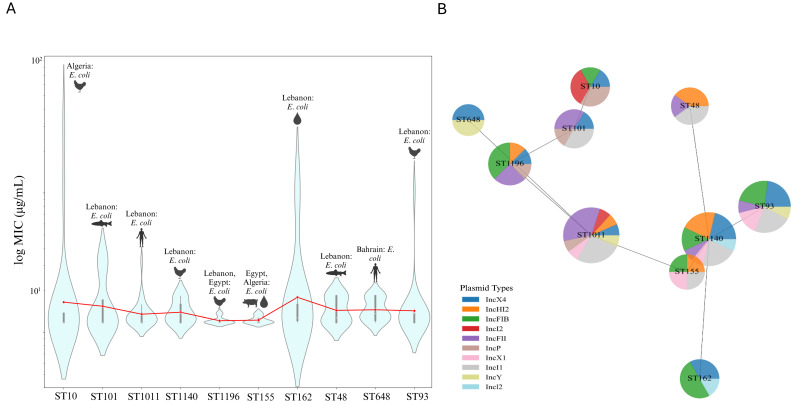
MIC variability and plasmid association among geographically distributed sequence types. (**A**) Violin plots illustrating the MIC variability among the top 10 most geographically distributed sequence types with available MIC data. The width of each violin represents the density of data points at different MIC values, with wider sections indicating a higher concentration of data points. The red line overlay shows the mean MIC values across the different STs, providing a comparative view of resistance levels among the sequence types. (**B**) Minimum spanning tree with nodes represented as pie charts, where each node corresponds to a sequence type. The segments within each pie chart indicate the relative frequency of different plasmid types associated with that ST. The distance between nodes reflects the genetic or epidemiological relatedness between sequence types, with closer nodes suggesting a higher degree of similarity based on shared plasmid profiles. Edited with BioRender.com.

The extensive distribution of sequence types such as ST10, ST101, and ST1011 across various Arab countries indicates their epidemiological significance. The geographical spread of these sequence types is only one aspect of understanding their impact. To gain deeper insight into the resistance profiles associated with these STs, it is crucial to examine the available data related to MIC variability. The violin plots, which display the top 10 most geographically distributed sequence types with available MIC data, illustrate that ST10 exhibits the most significant MIC variability, with the highest MIC value recorded at 96 µg/mL, particularly from Algeria’s poultry samples. While there are other high MIC values present in the dataset, their specific sequence types were not detailed in the studies. In contrast, ST155 and ST1196 demonstrate narrower MIC distributions and lower maximum MIC values, primarily originating from Lebanon, Egypt, and Algeria. ST162 and ST93 also show relatively high maximum MIC values, emphasizing substantial resistance variability, especially from Lebanon’s environmental and poultry sources. ST101 and ST1011 display maximum MIC values of 32 µg/mL, with ST1011 reported from clinical samples and ST101 from fish samples in Lebanon, reflecting the wide distribution of highly resistant strains from various sources. The red line plot overlay confirms that while mean MIC values differ across ST types, certain types like ST10, ST162, ST93, ST101, and ST1011 consistently exhibit higher resistance levels, highlighting the need for targeted interventions in regions where these STs are prevalent (Figure 4A). In addition to the widely distributed sequence types, it is important to acknowledge that certain less geographically prevalent STs, such as ST340, also demonstrate alarmingly high resistance levels. For instance, ST340, identified in *Klebsiella pneumoniae* from a poultry farm in the UAE, exhibits a maximum MIC value of 256 µg/mL [152]. Although this sequence type is not as widely distributed as ST10 or ST101, its presence in specific regions with such high resistance is alarming to necessitate vigilant surveillance to prevent their broader dissemination and impact on public health (Supplementary Figure S4).

The minimum spanning tree with pie charts further illustrates the distribution of the top 10 most distributed ST types with available MIC data and their associated top 10 frequently occurring plasmids. ST10 is predominantly associated with the IncI1 and IncI2 plasmids, while ST101, ST1196, and ST648 exhibit a more balanced plasmid distribution, including significant frequencies of IncX4. ST1011 and ST1140 show varied plasmid content, with eight and seven plasmids, respectively. ST155 has a more concentrated plasmid profile, with IncX1, IncHI2, IncFIB, and IncI1 being equally frequent. The proximity of ST types with similar plasmid profiles, such as ST10 and ST101, suggests potential genetic or epidemiological links (Figure 4B).

### 3.5. Phylogenetic Analysis of mcr-Bearing E. coli Isolates from Arab Countries

**Figure 5:**
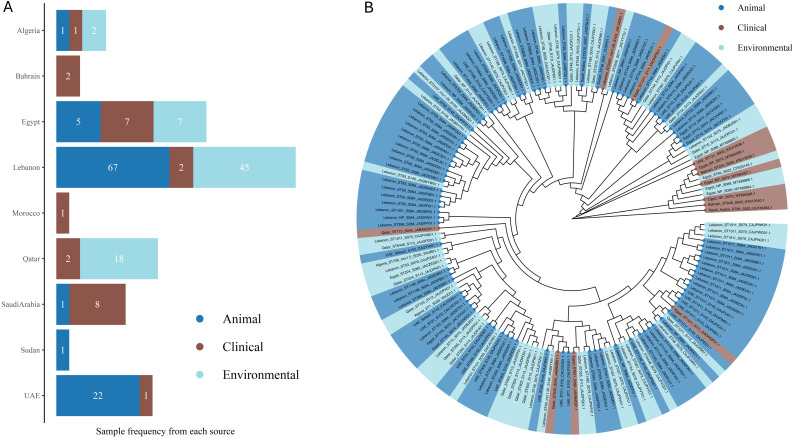
Phylogenetic relationships and source distribution of *E. coli* isolates from Arab countries. (**A**) The bar chart displays the frequency of samples from different sources across countries with available WGS data. Bars are colored according to source: animal (blue), clinical (brown), and environmental (light blue). Sample counts are shown inside the bars. (**B**) The tree was generated with IQ-TREE 2 using the Maximum Likelihood method. Branches are unscaled. Highlights indicate sources: animal (blue), clinical (brown), and environmental (light blue). Tip labels indicate the sample names as follows: Country_ST Type_*mcr* Type_Study Number_Accession Number. Only *mcr* types other than *mcr1* are written in the labels. NP for STs means not reported. Edited with BioRender.com.

Building on the observed distribution of sequence types across the Arab countries, we sought to investigate the genetic relationships among *mcr*-carrying isolates. We focused mainly on *E. coli* due to the limited availability of WGS data for other bacterial types. The number of isolates with available WGS data varies significantly between countries. Lebanon leads in animal isolates with 67 samples and, along with Qatar, provides substantial environmental data, with Lebanon contributing 45 samples and Qatar, 18. In contrast, countries like Algeria, Bahrain, Morocco, Sudan, and Saudi Arabia have fewer isolates represented, particularly in the environmental and clinical categories. Egypt offers a balanced contribution with five animal, seven clinical, and seven environmental isolates, while Saudi Arabia reports one animal, eight clinical, and no environmental isolates. Bahrain and Sudan each contribute only a small number of clinical and animal isolates, and Morocco provides a single clinical sample. This distribution indicates the varying levels of data available from different countries, with Lebanon contributing the most comprehensive datasets (Figure 5A).

Through a comprehensive phylogenetic analysis of the available WGS data, we visualized the evolutionary linkages and potential clonal expansion among *E. coli* isolates from various Arab countries, revealing significant close genetic relationships that suggest shared sources or transmission routes across different ecological niches and regions. For instance, the environmental isolate from Qatar and the animal isolate from the UAE (both ST1011) demonstrate a close phylogenetic relationship, indicating a shared lineage. Similarly, the clinical isolates from Egypt (ST not reported) and Bahrain (ST224) are closely related, suggesting potential regional transmission within clinical settings. The analysis also highlights the interconnectedness of resistance genes across sources, such as the close relationship between an animal isolate from Lebanon (ST752) and an environmental isolate from Qatar (ST34). Notably, the animal isolate from Lebanon (ST69, *mcr-1.26*) and the animal isolate from the UAE (ST69) demonstrate a close genetic relationship, indicating possible cross-border dissemination of these specific variants. Additionally, the environmental isolate from Algeria (ST708, *mcr-1.5*) and the environmental isolate from Lebanon (ST23) are closely related (Figure 5B). These findings emphasize the need for coordinated surveillance across multiple sectors to monitor and control the spread of colistin resistance in the Arab region.

### 3.6. Regional Patterns of Antimicrobial Resistance and Virulence Gene Distribution

**Figure 6:**
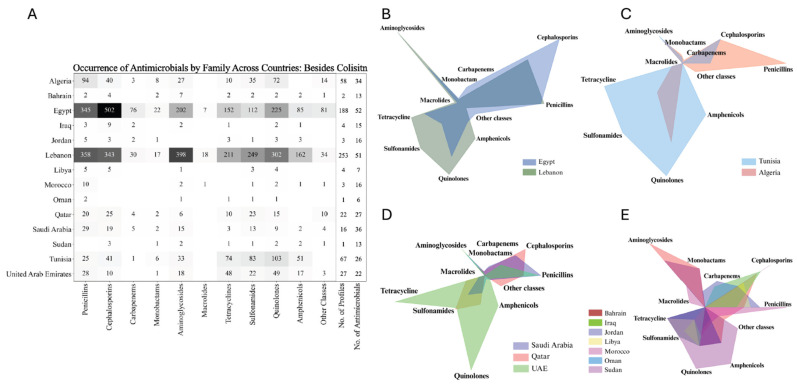
Antimicrobial resistance profiles across Arab countries: heatmap and comparative radar charts by antibiotic class. (**A**) Heatmap showing the total number of resistances to antimicrobials belonging to each family across various antibiotic classes for each Arab country. Darker shades represent a higher resistance to the respective antimicrobial family. (**B**–**E**) Radar charts displaying the distribution of antimicrobial resistance across different antibiotic classes for selected Arab countries. Each chart compares the resistance profiles among multiple countries, with each axis representing a different antibiotic class and the area within the chart reflecting the level of resistance in that class. Adjusted with BioRender.com.

The intricate relationship between sequence types, plasmid associations, and MIC variability provides a detailed view of how resistance mechanisms are distributed across different regions. However, understanding the full scope of antimicrobial resistance, phenotypically and genotypically, in these countries requires a broader examination of resistance patterns across multiple antibiotic classes. As the distribution and prevalence of these sequence types suggest potential regional resistance trends, it becomes essential to explore how these trends manifest in the actual resistance profiles observed in different countries. This analysis reveals significant differences in antibiotic resistance between key regions, particularly in Egypt and Lebanon, which stand out with higher resistance levels across various antibiotic classes, attributed to the larger number of profiles described from these regions (Figure 6A). Specifically, Lebanon exhibits higher resistance in aminoglycosides, macrolides, tetracyclines, quinolones, and amphenicols, while Egypt shows elevated resistance in cephalosporins, carbapenems, and monobactams. The differences in antimicrobial resistance between these two countries are statistically significant, highlighting distinct resistance patterns and challenges in each region (Figure 6B). Comparing Tunisia and Algeria also demonstrates significant differences, with Tunisia showing broader resistance across multiple antibiotic classes such as aminoglycosides and tetracyclines, while Algeria has concentrated resistance in specific antibiotics like penicillin and carbapenems (Figure 6C). With a limited number of profiles, comparisons between countries become more complex. However, an analysis of AMR profiles from Qatar, the UAE, and Saudi Arabia indicates distinct resistance trends. Isolates from Saudi Arabia exhibited increased resistance to penicillin, while isolates from Qatar showed elevated resistance to cephalosporins. In contrast, isolates from the UAE demonstrated heightened resistance to tetracyclines and a relatively more balanced resistance profile overall. These observations suggest varying resistance pressures and antibiotic usage practices across these countries (Figure 6D). The resistance profiles for the remaining countries—Bahrain, Iraq, Jordan, Libya, Morocco, Oman, Sudan, and Palestine—showed a high degree of similarity (Figure 6E). To obtain an overall insight, additional profiles and studies are needed for these countries. The occurrence of each antibiotic across all countries is comprehensively detailed in Supplementary Figure S5.

**Figure 7:**
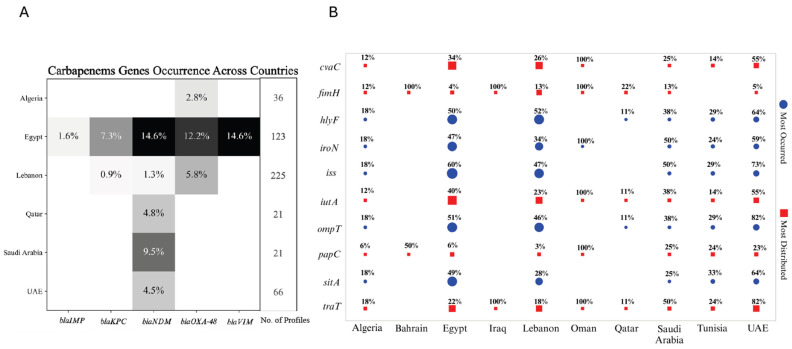
Distribution of carbapenemase and virulence genes across Arab countries: heatmap and bubble plot analysis. (**A**) Heatmap displaying the frequency of carbapenemase genes (*bla*_IMP_, *bla*_KPC_, *bla*_NDM_, *bla*_OXA-48_, *bla*_VIM_) across six Arab countries, with shading indicating the percentage of profiles in each country harboring these resistance genes. The number of profiles analyzed in each country is also indicated. No data were available from countries not mentioned in the heatmap. (**B**) Bubble plot illustrating the top 5 most frequently occurring (blue circles) and top 5 most widely distributed (red squares) virulence genes across various Arab countries. The size of the bubbles corresponds to the percentage of profiles in each country containing the respective virulence gene. The plot provides a comparative analysis of the prevalence and distribution of key virulence factors across different countries, normalized by the number of profiles in each region. Edited with BioRender.com.

The diversity and inconsistency in the nomenclature of resistance genes across various studies presented significant challenges in fully extracting and analyzing all resistance genes from the available data. This discrepancy in gene naming conventions often limited our ability to comprehensively assess the full spectrum of antimicrobial resistance. As a result, we decided to focus specifically on carbapenem resistance, given its critical importance and the more consistent reporting across studies. Egypt, with 123 profiles of antimicrobial-resistant genes, exhibits a notably high frequency of resistance, particularly for *bla*_NDM_ and *bla*_VIM_ (both at 14.6%) and *bla*_OXA-48_ (12.2%). This suggests a significant burden of carbapenem resistance despite a relatively smaller sample size compared to Lebanon. In contrast, Lebanon, with nearly double the number of profiles (225), shows much lower occurrences, with *bla*_OXA-48_ being the most frequent at just 5.8%, followed by *bla*_VIM_ (1.3%) and *bla*_NDM_ (0.9%). This disparity underscores Egypt as a potential hotspot for carbapenem resistance, while Lebanon, with its larger dataset, appears to have a comparatively lower prevalence. Saudi Arabia and Qatar, each with 21 profiles, also show occurrences of carbapenemase genes, particularly in Saudi Arabia with *bla*_NDM_ (9.5%). The UAE, with 66 profiles, demonstrates the presence of the resistance gene *bla*_NDM_ at 4.5%, whereas Algeria, with 36 profiles, presents the lowest overall frequencies. The absence of data or the small sample sizes from the remaining countries likely contributed to the negative or inconclusive results in those regions (Figure 7A).

While understanding resistance mechanisms, particularly carbapenem resistance, is crucial for addressing the public health threat posed by multidrug-resistant organisms, it is equally important to consider the role of virulence factors in the spread and severity of infections. These virulence genes contribute to the pathogenicity of bacteria, influencing their ability to cause disease and evade host defenses. The analysis of the top five most occurred and five most distributed virulence genes across various countries, normalized by the number of profiles in each region presented the following results. Egypt and Lebanon, with nearly equal profile counts (94 and 93, respectively), display distinct patterns. Egypt shows higher occurrences of most analyzed genes except for *fimH* (encodes a type 1 fimbrial adhesin involved in bacterial adhesion), where Lebanon has a higher occurrence. Both countries, however, exhibit almost similar levels for *hlyF* (encodes a hemolysin associated with intracellular bacterial survival and lysis of host cells) and *papC* genes (encodes a structural component of P pili, aiding in adhesion and colonization). Despite smaller sample sizes, countries like Algeria and Tunisia, with 17 and 21 profiles, respectively, display relatively high occurrences of several genes. Notably, 33% of profiles in Tunisia report the presence of *sitA* (encodes a transporter protein involved in iron acquisition), with 18% in Algeria, making it one of the most frequently reported genes. In Tunisia, genes like *ompT* (encodes an outer membrane protease involved in immune evasion), *iss* (encodes an increased serum survival protein that aids in resistance to host complement), and *hlyF* are each detected in 29% of profiles, while in Algeria, *ompT* and *iss* are present in 18%. Bahrain, with only two profiles, shows a 100% occurrence of *fimH* and a 50% occurrence of *papC*, though the small sample size necessitates cautious interpretation. The UAE, Qatar, and Saudi Arabia, with twenty-two, nine, and eight profiles, respectively, exhibited high occurrences of genes. In Qatar, *fimH* is prevalent, while in Saudi Arabia, *iroN* (encodes a siderophore receptor involved in iron uptake), *iss*, and *traT* (encodes a surface exclusion protein contributing to serum resistance) are prominent. The UAE exhibits an elevated presence of *ompT* and *traT*, with 82% of profiles showing these genes, suggesting a significant distribution within the country. Countries with very minimal profiles, such as Oman and Iraq, also show high occurrences of specific genes, like *fimH* and traT in both of the countries, and *iutA* (encodes an aerobactin siderophore receptor aiding in iron acquisition) in Oman only, each with 100% occurrence. However, these findings may be less representative due to the limited sample sizes. Overall, the data highlight regional differences in the prevalence and distribution of these virulence genes. Egypt and Lebanon emerge as key regions for the study of these factors, while smaller sample sizes from other countries provide valuable, though potentially less representative, insights (Figure 7B). A comprehensive heatmap displaying the distribution of all virulence genes across countries is provided in Supplementary Figure S6.

### 3.7. Risk of Bias Analysis

The risk of bias analysis was conducted across various studies, numbered along the x-axis similar to the numbering of Supplementary File S1, with several domains of bias represented on the y-axis. Each domain—Selection Bias, Performance Bias, Detection Bias, Attrition Bias, and Reporting Bias—is color-coded to indicate the level of bias risk: green for low risk, yellow for moderate risk, and red for high risk. From the figure, it is evident that Selection Bias and Performance Bias are frequently encountered across the studies, with notable instances of high-risk (red) and moderate risk (yellow) interspersed among low-risk studies (green). Detection Bias also shows a mix of risks but is predominantly low risk. Attrition Bias and Reporting Bias are mostly green, suggesting low risk across the majority of studies; however, there are occasional instances of moderate ROB. Notably, certain sets of studies show higher instances of bias in specific domains, indicating potential areas where methodological improvements are needed. For example, studies between numbers 20 and 40 show significant instances of high and moderate Selection and Performance Bias, highlighting a need for better randomization and blinding in those studies (Supplementary File S2). Overall, the majority of studies exhibit a low risk of bias upon analysis.

## 4. Discussion

This review describes the prevalence and distribution of mobilized colistin resistance genes in animal, clinical, and environmental isolates across Arab countries. Taking into account the unique healthcare infrastructure, agricultural practices, and regulatory frameworks in the Arab world, we emphasize the critical need for region-specific surveillance and intervention strategies to address the growing issue of colistin resistance.

The distribution of *mcr* genes in animal sources in Egypt indicates the critical role of livestock as reservoirs for antimicrobial-resistant genes. The widespread and unregulated use of antibiotics in poultry and other livestock has significantly contributed to the spread of *mcr* genes [175]. Beyond the *mcr-1* gene, other variants such as *mcr-2*, *mcr-3*, and *mcr-4* have been identified in *E. coli* isolates from raw milk and mastitis samples (Figure 2A and Figure 3). Notably, the *mcr-7* gene was recently reported in *P. aeruginosa* isolates from clinical mastitic milk samples in Egypt, marking the first detection of this gene in such isolates [50]. The high frequency of *mcr*-positive isolates from animal sources supports the established correlation between intensive antibiotic use in agriculture and the prevalence of these resistance genes [11]. The diverse presence of *mcr* genes suggests multiple independent acquisitions, emphasizing the urgent need for comprehensive surveillance and stringent antibiotic stewardship policies. In Lebanon, environmental surveillance has been pivotal in detecting *mcr* genes (Figure 3). Studies by Hmede et al. and Sourenian et al. reported the presence of *mcr-1* in irrigation water and the Mediterranean Sea [82,86], as well as in *Proteus mirabilis* isolated from drinking water consumed by Syrian refugees in different camps [87]. The relatively higher number of environmental isolates in Lebanon may reflect either more effective monitoring practices or higher levels of environmental contamination, emphasizing the need for improved waste management and water treatment strategies. In addition to the Arab countries, the global dissemination of colistin-resistant *E. coli* exhibits the highest prevalence in Asia, Africa, and Latin America, where approximately 25% of isolates from the studies are resistant to colistin. This stark contrast with the much lower rates observed in Europe and North America is primarily due to the widespread use of colistin in livestock in these regions, coupled with insufficient regulatory measures. In contrast, Europe and North America have implemented more stringent controls, resulting in lower prevalence rates. The high resistance levels in developing countries are further exacerbated by inadequate monitoring systems and poor biosecurity measures in food production. This resistance is not confined to livestock but extends through the food chain, impacting meat, eggs, and dairy products [176]. The focus on environmental sources aligns with Bardet et al., who highlighted the importance of environmental monitoring in identifying the spread of AMR genes through water and soil [177].

Algeria demonstrates a relatively balanced distribution of *mcr* genes across various environmental and animal sources, including sperm culture, urine, sea water, fresh stool, chicken farms, slaughterhouses, agricultural soil, well water, migratory birds, fresh vegetables, and ready-to-eat samples. The presence of colistin-resistant *Klebsiella pneumoniae* with *mcr-8*, *E. coli* isolates with *mcr-3*, *mcr-1.5*, and *Citrobacter gilardii* harboring *mcr-5.1* from these sources reflects the diverse selection pressures exerted on bacterial populations in different environments, driving the acquisition of these genes (Figure 3). The observed variety of *mcrs* also suggests that the environmental, agricultural, and clinical settings in Algeria contribute uniquely to the spread and maintenance of colistin resistance. This highlights the importance of understanding the specific selective pressures that facilitate the persistence and dissemination of *mcr* genes, as this knowledge is crucial for developing targeted surveillance and intervention strategies [13]. Tunisia, similar to Egypt, focuses on screening animal sources for *mcr* genes but on a smaller scale. The high prevalence of *mcr-1* in *E. coli* from broiler chickens points to extensive colistin use in the poultry industry (Figure 3), with *mcr-2* detection indicating broader resistance. The narrower range of *mcr* variants compared to Egypt may be due to less intensive surveillance or genuinely lower diversity, highlighting the need for expanded surveillance to fully address antimicrobial resistance [132,178].

Studies from Saudi Arabia focus primarily on clinical sources, highlighting significant challenges with *mcr* genes in healthcare settings. The detection of *mcr-8* and *mcr-4* in clinical isolates underlines the need for rigorous monitoring to prevent the spread of these resistant strains. Li et al. (2017) identified clinical environments as hotspots for highly resistant *mcr*-positive strains, necessitating infection control measures and antibiotic stewardship programs [179]. In contrast, Qatar and the UAE report various *mcr* gene types mainly from animal sources (Figure 3), indicating the role of imported livestock and animal products in disseminating antimicrobial resistance. The lack of data on environmental sources points to critical gaps in AMR surveillance, emphasizing the necessity for a comprehensive One Health approach that integrates human, animal, and environmental health. Countries with diverse animal product sources often report varying *mcr* gene prevalence, stressing the importance of comprehensive surveillance systems to effectively tackle AMR [11].

The detection of *mcr-10* in *E. coli* from chicken samples in Jordan suggests the presence of a unique plasmid-born colistin-resistant gene, stressing the urgent need for further study and characterization (Table 1 and Figure 3) [180]. Despite the high prevalence of *mcr-10* in Asian and European countries, this is the first reported case from the Arab world after detecting it in Egypt from raw milk samples [51,159]. This might be attributed to either insufficient sampling or sampling bias [181]. Smaller-scale studies in Sudan, Libya, Morocco, Bahrain, and Palestine reveal a mix of reservoirs, although limited data suggest lower prevalence or data gaps. The presence of *mcr-8* in clinical sources in Morocco indicates focused clinical surveillance but highlights the need for broader environmental and animal source monitoring. These findings resonate with filling data gaps to understand the complete epidemiology of *mcr* genes (Figure 2A and Figure 3) [11].

The surveillance of resistance genes and a detailed understanding of their distribution and impact, when carried by particular sequence types, are part of facing the challenge of antimicrobial resistance. The presence and widespread distribution of specific STs within the Arab region, particularly ST101, ST1011, and ST10 (Supplementary Figure S3), align with global trends in the spread of multidrug-resistant bacteria. ST101, frequently associated with the presence of New Delhi metallo-beta-lactamase (*bla*_NDM_) and other resistance mechanisms, has been reported across Asia and Europe [182]. Its presence in multiple Arab countries, including Algeria, the UAE, Egypt, and Lebanon, indicates the regional impact of this sequence type. However, the studies we obtained from Arab countries did not report the presence of *bla*_NDM_ in ST101, which may reflect a lack of comprehensive data from the entire region. ST1011, observed in clinical settings, carries *bla*_NDM_ and exhibits carbapenem resistance along with colistin, making it a critical concern in the region [183]. Despite being less common worldwide, ST1011 is one of the most prevalent and occurring STs in Arab countries, according to our findings. Additionally, ST10 emerged as the most widely distributed sequence type across the Arab countries, consistent with global reports of its extensive presence in diverse environments. The significant distribution of ST10 in our study aligns with findings that emphasize its adaptability and resilience, particularly in agricultural settings and human microbiomes. Its association with the *mcr-1* gene further underscores ST10’s critical role in the broader context of antimicrobial resistance [183,184].

To deepen our understanding of the dissemination of *mcr* genes across Arab countries, it is equally important to examine the diversity and resistance profiles of these sequence types. Analyzing their distribution across different countries not only enhances our comprehension of antimicrobial resistance but also uncovers the complex regional factors that drive its spread throughout the Arab world. For instance, in Saudi Arabia, the ST68 strain of *E. coli*, isolated from blood, exhibits the most extensive resistance profile among the studied countries, with an MIC of 16 µg/mL, indicating a high level of resistance taken into account the presence of the *bla*_NDM_ gene, which confers resistance to carbapenems [133]. This extensive resistance profile may be attributed to research targeted at high-risk or high-prevalence areas, unique health and demographic factors such as a large expatriate population, frequent international travel for religious pilgrimages, and high urbanization rates. Additionally, environmental conditions like high temperatures and arid climates play a role in the persistence and transmission of resistant bacteria. Similarly, in Algeria, ST10 isolates from poultry samples showed high MIC variability and maximum MIC values (96 µg/mL) among the top 10 distributed STs (Figure 4A), suggesting substantial resistance levels, which is an indicator of the extensive misuse of antimicrobials in animal husbandry, similar to other Arab countries [113]. ST155 and ST1196 isolates from Lebanon, Egypt, and Algeria show narrower MIC distributions and lower maximum MICs compared to ST10. The high prevalence of *mcr-1* and other resistance genes in these isolates indicates significant clinical treatment challenges and intensive antibiotic use in poultry farming [97].

To further analyze the implications of these findings, the minimum spanning tree with pie charts provides a comprehensive view of the distribution of the top 10 ST types (Figure 4B) with available MIC data and their associated plasmids. ST10 is predominantly associated with IncI1 and IncI2 plasmids—known vectors for *mcr* genes. These plasmids play a crucial role in spreading colistin resistance. A study also identified these plasmids in ST10 strains, highlighting their role in spreading colistin resistance. The identification of ST10 isolates carrying IncI1 and IncI2 plasmids is significant due to the high transmissibility of these plasmids, which facilitate the spread of antibiotic resistance genes such as *bla*_CTX-M-55_ and *mcr-1* across different various strains. This poses an epidemiological concern as ST10 is widely disseminated in both humans and animals, indicating a potential for zoonotic transmission [185,186]. The acquisition of multiple plasmids suggests different resistance mechanisms and indicates rapid acquisition and dissemination of resistance genes. Understanding genetic linkages between ST types with similar plasmid profiles is essential for developing effective control strategies.

The analysis of close genetic relationships between isolates from different ecological niches, such as animals, the environment, and clinical settings, strongly suggests shared sources or transmission routes across borders, facilitated by factors like trade, travel, and common agricultural practices. For instance, the close genetic ties between isolates from Qatar and the UAE (ST1011) and between Lebanon and the UAE (ST69) indicate the possible cross-border dissemination of these resistant strains, a pattern that has been similarly observed in studies documenting the spread of AMR across different regions due to the global movement of livestock, food products, and human populations [187]. This cross-border dissemination is particularly concerning given the potential for clonal expansion of specific *mcr*-bearing *E. coli* lineages, which could result in widespread resistance across multiple countries. The close relationship between clinical isolates from Egypt and Bahrain, despite the different STs reported, suggests that healthcare settings in these countries may be linked through the movement of patients, aligning with reports indicating the role of healthcare-associated infections in the spread of AMR [187]. Moreover, the interconnectedness of resistance genes across different sources, as seen in the phylogenetic relationship between an animal isolate from Lebanon (ST752) and an environmental isolate from Qatar (ST34), further supports the idea that resistance genes can move between ecological niches, potentially facilitated by environmental reservoirs such as contaminated water and soil (Figure 5B) [188]. Expanding the availability of WGS data across the region would provide a more comprehensive understanding of the transmission dynamics of *mcr*-bearing *E. coli* and other strains, which would help to inform more effective public health responses. Given the potential for the rapid dissemination of these resistant strains across borders, international cooperation and collaboration are essential to effectively manage and mitigate the threat of colistin resistance in the Arab region and beyond.

Building on the examination of sequence types and their associated resistance mechanisms, it is evident that the patterns of antimicrobial resistance vary significantly across the Arab region. Egypt and Lebanon exhibit higher resistance in several antibiotic classes compared to other countries. According to the literature, the environmental and clinical isolates from Lebanon carry multiple resistance genes, indicating extensive antibiotic use and the resulting selective pressure [98]. In Egypt, elevated resistance is observed in cephalosporins and carbapenems. One possibility for the higher rates of carbapenem resistance in Egypt is the widespread inappropriate and excessive use of broad-spectrum antibiotics, particularly in hospital settings. Another reason could be the significant presence of extended-spectrum beta-lactamase (ESBL)-producing Enterobacterales, which reduces the effectiveness of many antibiotics and increases the reliance on carbapenems, further driving resistance. Lastly, we cannot exclude the possibility that the data might be insufficient or underreported in other countries, which could result in an underestimation of carbapenem resistance levels elsewhere, making Egypt’s resistance rates appear disproportionately high in comparison [56]. Tunisia shows a broader resistance across multiple antibiotic classes compared to Algeria, such as aminoglycosides and tetracyclines, while Algeria demonstrates concentrated resistance in specific antibiotics like antibiotics belonging to penicillin. The broader resistance in Tunisia could indicate more extensive or varied antibiotic usage practices. Algeria’s concentrated resistance reflects targeted usage or higher reliance on specific antibiotic classes similar to Egypt, driven by local prescribing practices or availability (Figure 6A–E and Figure 7A). The decreased amount of resistance data from the other Arab countries hinders comprehensive analysis and accurate comparisons.

The observed patterns of antimicrobial resistance not only highlight differences in antibiotic usage and healthcare practices but also correlate with variations in the distribution of virulence genes across the Arab region. These regional differences in virulence gene distribution further reflect the complex interactions of local environmental factors, healthcare practices, and bacterial genetic diversity. Egypt, with a higher occurrence of most analyzed genes except *fimH*, where Lebanon is more prevalent, may be influenced by differing antibiotic usage and infection control measures, consistent with the results of detecting different AMR profiles in different regions. In Saudi Arabia, Qatar, and the UAE, the prominent presence of genes like *iroN*, *iss*, *traT*, and *ompT* suggests significant regional variations in bacterial populations, potentially driven by local healthcare practices and selective pressures such as specific antibiotic use, chemical exposure, or environmental stress. Similarly, Tunisia and Algeria’s high occurrences of genes like *sitA*, *ompT*, and *iss* further highlight how specific environmental and clinical conditions may favor certain virulence factors (Figure 7B). Rossi et al. support these observations by noting that the number and diversity of virulence genes can vary significantly between regions, with differences in antibiotic usage being a key driver of these variations [189].

## 5. Conclusions

The spread of *mcr* genes in the Arab world is driven by multiple factors, including the widespread use of antibiotics in agriculture, the importation of food products containing antimicrobial-resistant bacteria, and increased cross-border travel, which facilitates the movement of resistant strains. The high population density in some regions creates additional challenges for public health systems, where the risk of transmission is amplified in urban areas and healthcare facilities. Within the healthcare sector, insufficient infection control practices, lack of antibiotic stewardship programs, and inadequate surveillance systems contribute to the proliferation of resistant bacteria, making it difficult to contain outbreaks. In regions affected by various socio-economic challenges, including regional conflicts, the situation is worsened by disruptions in infrastructure and public health services.

However, this study faces several limitations. First, the data availability varies significantly across the Arab world; while some countries have abundant data, others provide very limited information, and certain parts of the Arab regions lack data entirely. This disparity in data hinders a comprehensive regional understanding. Second, some studies used PCR-based detection methods for *mcr* genes, which may require further confirmation through advanced techniques like sequencing, but the lack of comprehensive data pushed to include them in the analysis. Third, the diverse socio-political and economic situations across Arab countries posed a challenge for data collection, with conflicts in some areas and rapid development in others, making it difficult to analyze all contributing factors to *mcr* gene spread consistently. Additionally, another limitation is the lack of unified surveillance systems across the regions, which hampers efforts to monitor and respond to antimicrobial resistance trends effectively.

Despite varying levels of research and surveillance across the region, many areas still have significant gaps in data, limiting the ability to fully understand and address the problem. Filling these gaps with coordinated research efforts, along with strengthening healthcare systems, regulating antibiotic use in agriculture, and improving international collaboration, will be crucial in mitigating the spread of *mcr* genes and combating antimicrobial resistance on both a regional and global scale.

## Data Availability

The data presented in this study are available on request from the corresponding author.

## Supplementary Materials

The following supporting information can be downloaded at: https://www.mdpi.com/article/10.3390/antibiotics13100958/s1. Figure S1: Yearly Rate of *mcr* Gene Publications Across 15 Arab Countries (2016–2024): This figure displays the yearly rate of publications reporting the presence of *mcr* genes in 15 Arab countries from 2016 to 2024. Each panel (A–O) represents a different country, with the x-axis corresponding to the years and the y-axis indicating the number of publications per year. The connected data points illustrate trends in research activity over time. For example, Panel C (Egypt) shows a peak in publications around 2021. The *mcrs* annotation reflects the detection of the gene in the respective year. Figure S2: Distribution of the Top 10 Occurred and Top 10 Distributed Sequence Types Across 12 Arab Countries: This figure illustrates the distribution of the top 10 most frequently occurring and top 10 most geographically distributed sequence types (STs) across 12 Arab countries. Each row represents a specific sequence type, while the columns represent different countries. The percentages indicate the proportion of reports for each sequence type within each country, reflecting how widely these STs are distributed or how frequently they occur in the reported studies. For instance, ST101 shows significant prevalence in several countries, with the highest percentage in Lebanon, while ST10 exhibits broad geographic distribution with varying frequencies across multiple countries. Figure S3: Top Three Strains distribution per country: Network graph showing the distribution of the three most prevalent and widely distributed sequence types (ST10, ST101, ST1011, ST10) across the Arab countries. The graph displays the percentage of each sequence type found in each country. Countries not represented in the graph indicate that these sequence types were not reported there. Edited with BioRender.com. Figure S4: Distribution of Minimum Inhibitory Concentration Values by Sequence Type Across Arab Countries: This figure presents box plots illustrating the distribution of minimum inhibitory concentration values for different sequence types reported across Arab countries. Each box plot represents the MIC values associated with a particular sequence type, with the x-axis listing the sequence types and the y-axis showing the MIC values on a logarithmic scale. The boxes indicate the interquartile range, with the line inside each box representing the median MIC value. The whiskers extend to the smallest and largest values within 1.5 times the interquartile range, while outliers are plotted as individual points. Figure S5: Antimicrobial Occurrence Across Arab Countries (Excluding Colistin): This figure illustrates the occurrence of various antibiotics resistant across different Arab countries, excluding colistin. Each row represents a specific country, while each column represents a different antibiotic. The numbers within the cells indicate the number of occurrences or reports for each antibiotic in the corresponding country, while the shading intensity reflects the frequency, with darker shades indicating higher occurrence. Figure S6: Virulence Gene Occurrence Across Isolates from The Arab Countries: This figure presents the occurrence of various virulence genes across different Arab countries. Each row corresponds to a specific country, while each column represents a different virulence gene. The numbers within the cells indicate the number of occurrences or reports for each virulence gene in the respective country, with darker shading reflecting higher frequencies. File S1: The supplementary file contains several sheets detailing different aspects of the systematic review process. The first sheet, “Searching Strategy”, outlines the search strategy used to gather relevant papers for the review. The second sheet, “Title and Abstract Filtration”, describes the filtering process based on titles and abstracts. The third sheet, “Full Text Filtration”, presents the criteria used for filtering papers at the full-text stage. In the fourth sheet, “Filtered Papers”, the final list of selected papers after filtration is provided. The fifth sheet, “Table of Extracted Data”, contains the extracted data from these papers in a structured table format. Finally, the sixth sheet, “ROB (Risk of Bias)”, assesses the risk of bias in the studies included in the systematic review. File S2: Risk of Bias Analysis: This interactive figure allows for a detailed examination of potential biases across the studies included in the review. The figure is designed to be user-friendly, enabling you to hover over and click on different elements to explore specific areas of bias, such as selection, performance, detection, attrition, and reporting bias. Color coding is used to indicate the level of bias: green for low risk, yellow for moderate risk, and red for high risk. File S3: Alluvial Diagram of Sequence Type Distribution Across Arab Countries: this figure represents the flow and distribution of sequence types across various Arab countries. In this diagram, each stream represents a specific sequence type, with the width of the stream corresponding to the frequency or number of occurrences of that sequence type in each country. The streams flow horizontally, crossing through different countries, indicating the presence and distribution of the sequence types. Wider streams signify higher occurrences, while intersections of streams highlight where sequence types are shared between countries.
