# Supplementary material for: A Systematic Review and Comprehensive Analysis of mcr Gene Prevalence in Bacterial Isolates in Arab Countries
Source: Antibiotics (Basel). 2024 Oct 11;13(10):958. doi: 10.3390/antibiotics13100958 (PMC11505126; doi:10.3390/antibiotics13100958)
Supplement: Supplementary file 1 [file antibiotics-13-00958-s001.zip › antibiotics-3193889-supplementary Figures.pdf]

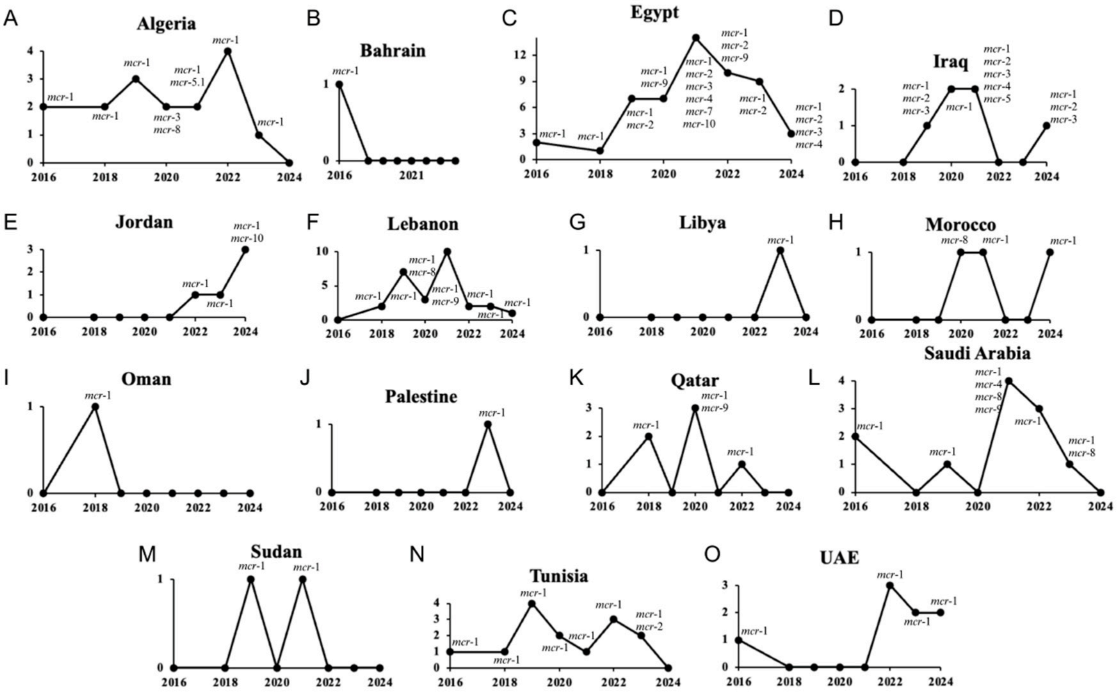

Figure S1. Yearly rate of mcr gene publications across 15 Arab countries (2016–2024). This figure displays the yearly rate of publications reporting the presence of *mcr* genes in 15 Arab countries from 2016 to 2024. Each panel (A–O) represents a different country, with the x-axis corresponding to the years and the y-axis indicating the number of publications per year. The connected data points illustrate trends in research activity over time. For example, Panel C (Egypt) shows a peak in publications around 2021. The *mcr* annotation reflects the detection of the gene in the respective year.

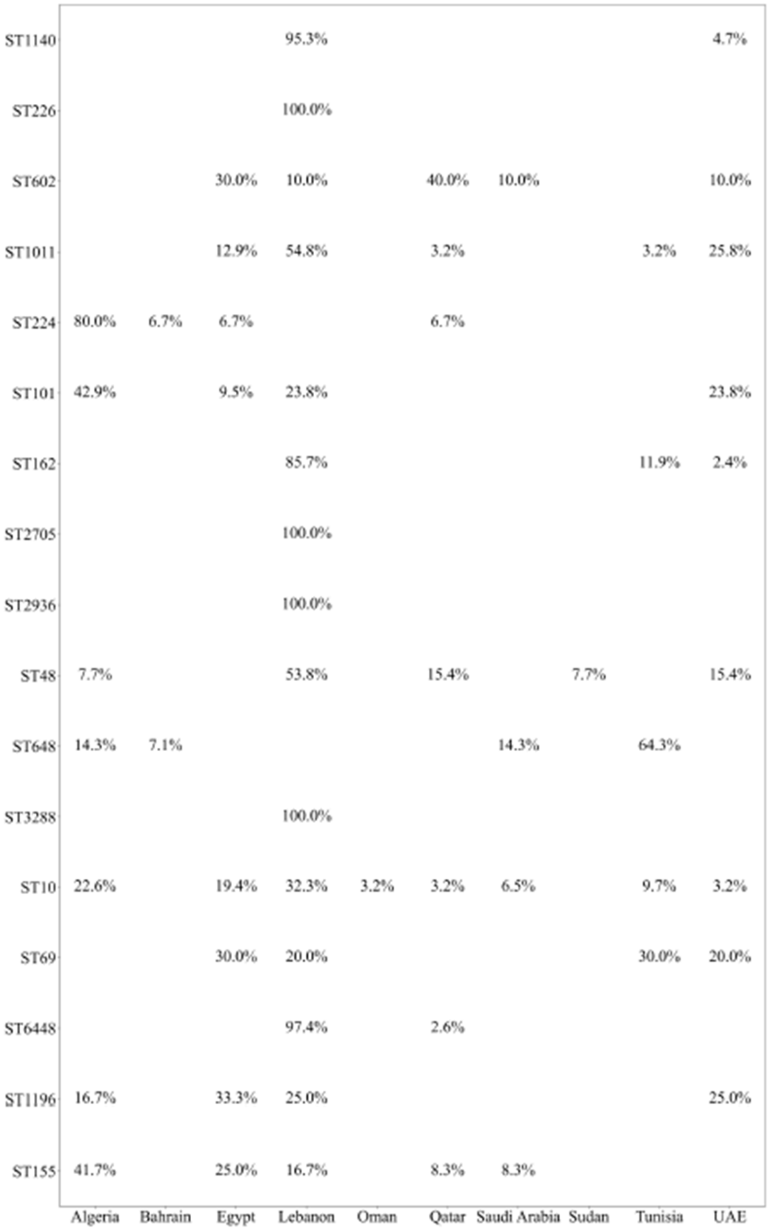

Figure S2. Distribution of the top 10 occurred and top 10 distributed sequence types across 12 Arab countries. This figure illustrates the distribution of the top 10 most frequently occurring and top 10 most geographically distributed sequence types (STs) across 12 Arab countries. Each row represents a specific sequence type, while the columns represent different countries. The percentages indicate the proportion of reports for each sequence type within each country, reflecting how widely these STs are distributed or how frequently they occur in the reported studies. For instance, ST101 shows significant prevalence in several countries, with the highest percentage in Lebanon, while ST10 exhibits a broad geographical distribution with varying frequencies across multiple countries.

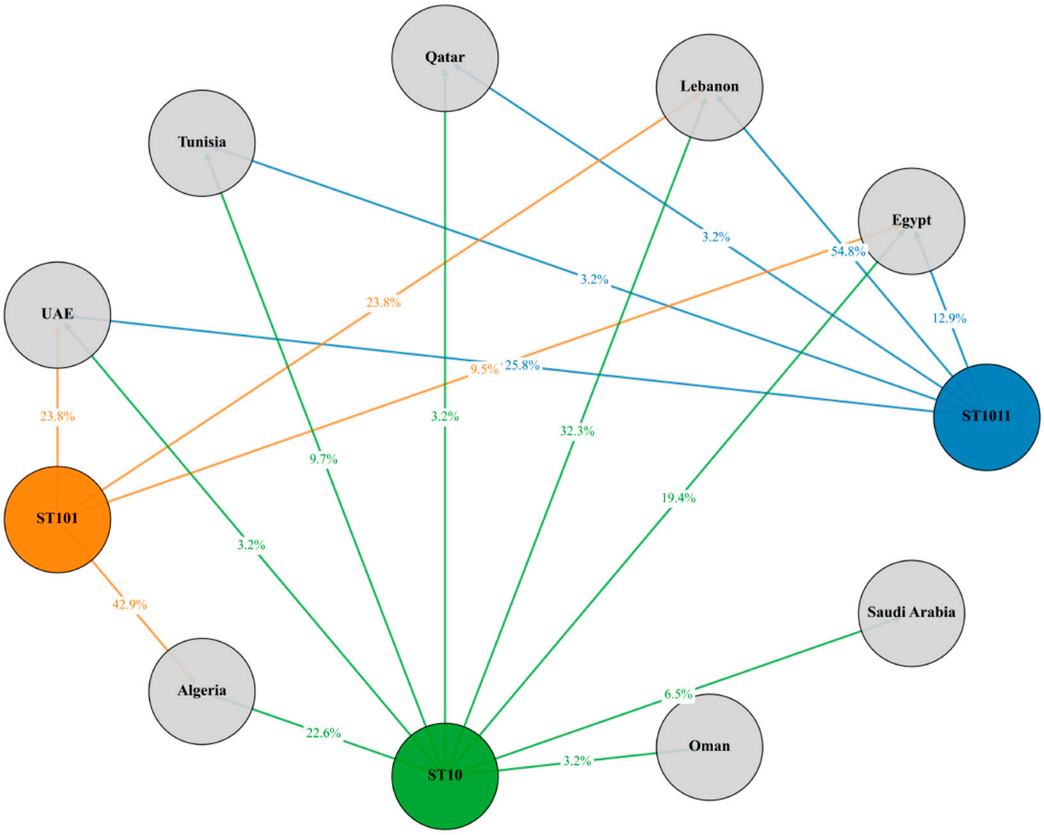

Figure S3. Top three strain distribution per country. Network graph showing the distribution of the three most prevalent and widely distributed sequence types (ST10, ST101, ST1011, ST10) across the Arab countries. The graph displays the percentage of each sequence type found in each country. Countries not represented in the graph indicate that these sequence types were not reported there. Edited with BioRender.com.

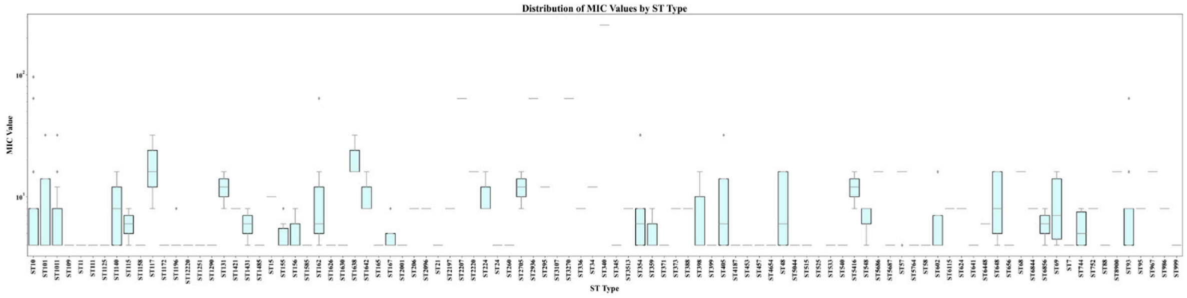

Figure S4. Distribution of minimum inhibitory concentration (MIC) values by sequence type across Arab countries. This figure presents box plots illustrating the distribution of minimum inhibitory concentration values for different sequence types reported across Arab countries. Each box plot represents the MIC values associated with a particular sequence type, with the x-axis listing the sequence types and the y-axis showing the MIC values on a logarithmic scale. The boxes indicate the interquartile range, with the line inside each box representing the median MIC value. The whiskers extend to the smallest and largest values within 1.5 times the interquartile range, while outliers are plotted as individual points.

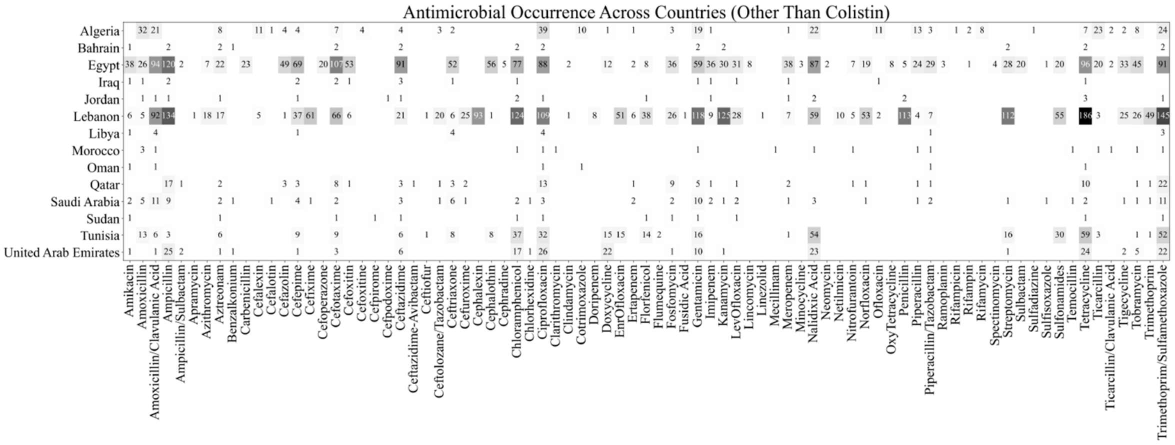

Figure S5. Antimicrobial occurrence across Arab countries (excluding colistin). This figure illustrates the occurrence of various types of antibiotic resistance across different Arab countries, excluding colistin. Each row represents a specific country, while each column represents a different antibiotic. The numbers within the cells indicate the number of occurrences or reports for each antibiotic in the corresponding country, while the shading intensity reflects the frequency, with darker shades indicating a higher occurrence.

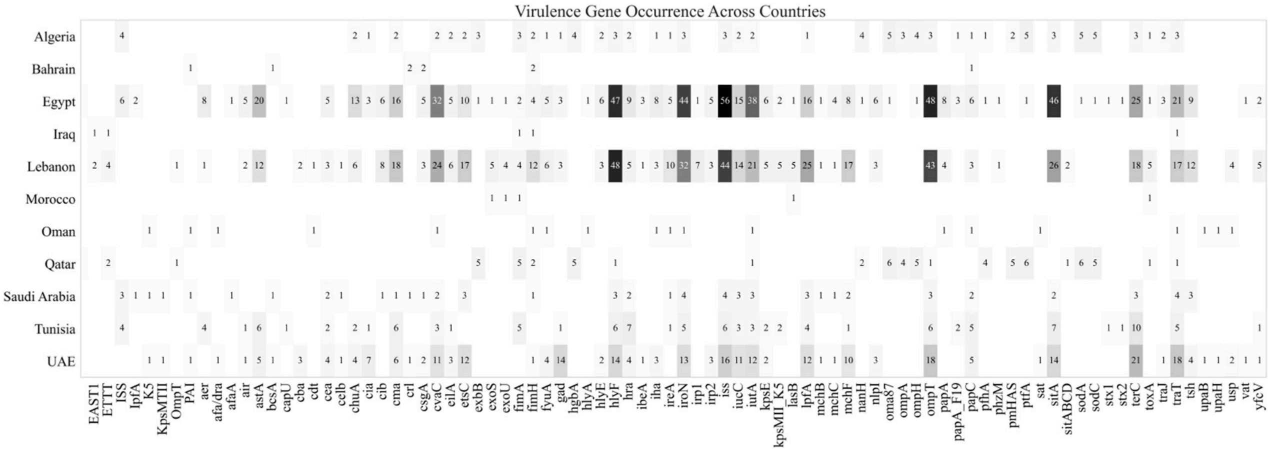

Figure S6. Virulence gene occurrence across isolates from the Arab countries. This figure presents the occurrence of various virulence genes across different Arab countries. Each row corresponds to a specific country, while each column represents a different virulence gene. The numbers within the cells indicate the number of occurrences or reports for each virulence gene in the respective country, with darker shading reflecting higher frequencies.

Supplementary File S2: Risk of Bias Analysis: This interactive figure allows for a detailed examination of potential biases across the studies included in the review. The figure is designed to be user-friendly, enabling you to hover over and click on different elements to explore specific areas of bias, such as selection, performance, detection, attrition, and reporting bias. Color coding is used to indicate the level of bias: green for low risk, yellow for moderate risk, and red for high risk.

Supplementary File S3: Alluvial Diagram of Sequence Type Distribution Across Arab Countries: this figure represents the flow and distribution of sequence types across various Arab countries. In this diagram, each stream represents a specific sequence type, with the width of the stream corresponding to the frequency or number of occurrences of that sequence type in each country. The streams flow horizontally, crossing through different countries, indicating the presence and distribution of the sequence types. Wider streams signify higher occurrences, while intersections of streams highlight where sequence types are shared between countries.
